# A model for analysis, systemic planning and strategic synthesis for health science teaching in the Democratic Republic of the Congo: a vision for action

**DOI:** 10.1186/1478-4491-2-16

**Published:** 2004-12-07

**Authors:** Florence Parent, Gérard Kahombo, Josué Bapitani, Michèle Garant, Yves Coppieters, Alain Levêque, Danielle Piette

**Affiliations:** 1 Department of Epidemiology and Health Promotion, School of Public Health, Université Libre de Bruxelles (ULB), Brussels, Belgium; 2 CREFSS-c/o Ministry of Health, Kinshasa/Gombe, Democratic Republic of the Congo; 3 Centre de Pédagogie Universitaire, Université Catholique de Mons (FUCAM), Belgium

## Abstract

**Background:**

The problem of training human resources in health is a real concern in public health in Central Africa. What can be changed in order to train more competent health professionals? This is of utmost importance in primary health care.

**Methods:**

Taking into account the level of training of secondary-level nurses in the Democratic Republic of the Congo (DRC), a systemic approach, based on the PRECEDE PROCEED model of analysis, led to a better understanding of the educational determinants and of the factors favourable to a better match between training in health sciences and the expected competences of the health professionals. This article must be read on two complementary levels: one reading, focused on the methodological process, should allow our findings to be transferred to other problems (adaptation of a health promotion model to the educational sphere). The other reading, revolving around the specific theme and results, should provide a frame of reference and specific avenues for action to improve human resources in the health field (using the results of its application in health science teaching in the DRC).

**Results:**

The results show that it is important to start this training with a global and integrated approach shared by all the actors. The strategies of action entail the need for an approach taking into account all the aspects, i.e. sociological, educational, medical and public health.

**Conclusions:**

The analysis of the results shows that one cannot bring any change without integrated strategies of action and a multidisciplinary approach that includes all the complex determinants of health behaviour, and to do it within the organization of local structures and institutions in the ministry of health in the DRC.

## Background

A partnership of the Ministry of Health of the Democratic Republic of the Congo (DRC) – more specifically, the directorate that is in charge of health science education – the French-speaking community of Belgium and various education and training associations made it possible to set up and carry out a teaching innovation project to bolster human resources in the health sector. One of the major public health challenges in Africa is to find efficient ways to enhance human resources in the health sector.

The goal of the medical policy in the DRC is to promote the health of the population by providing high-quality health care that is complete and integrated and in which the community participates, within the general context of the fight against poverty [1]. With this intention, the Ministry of Health defined six strategic axes to support the reinforcement of primary health care:

• restructuring the health system according to political, legislative and administrative orientations as well as updating standards of services;

• increasing the availability of resources by implementing an adequate administrative process;

• establishing an integrated system of preventive and curative care and health promotion for the target groups;

• strengthening the programmes of support to health activities;

• coordinating, promoting intrasectoral and intersectoral collaboration and partnership for health;

• promoting a suitable environmental framework for health.

The Ministry attaches priority importance to delivering high-quality care and health services and by: reaffirming the strategy of primary health care (PHC) as a fundamental option of the national policy on health; reaffirming the health zones' or districts' achieving a minimum package of activities as an operational unit; and regular procurement of essential drugs, including biological products and other laboratory reagents.

Training of nurses in the DRC is organized in all provinces of the country and conducted through technical medical institutes (ITM), medical educational institutes (IEM) for the secondary level and higher institutes of medical technology (ISTM) for the higher level. In 1998 there were 308 ITM and ISTM in the country; to date there are 254 schools of nurses (male and female) at the secondary level in the country recognized by the DRC government.

According to the type of management, these institutions are classified as public, officially agreed and private schools. However, the autonomy of management for all these schools is extensive, given the near inexistence of government subsidy. The infrastructure and quality of training differ widely from one ITM to another.

As a rule, the solutions that one observes in public health are located in the area of further training for health professionals. To the extent that further training is important, it is disappointing to ascertain the low yields that these various training courses have in improving the quality of health care and services [2]. There is a lack of prior analysis of the main training needs when it comes to developing abilities and independence, a lack of these training programmes' integration into existing structures, and a lack of consistency between these schemes and a complex environment composed of many interacting players. Moreover, it is difficult, for many different reasons, to escape from the many vertical programmes (more than 40 in the DRC) that impose their own training modules on target audiences that have been set beforehand on their level.

When it comes to basic training, the young nurses who have just graduated from secondary school and make up the critical mass of health professionals in primary health care are commonly required by specific private or church employers to enrol in a full year of training after their academic studies in order to try to fill the gaps between their basic training and health professionals' actual training needs. Confusion on the part of the ministries and other forces involved is attested to by the absence of vision and lack of expertise in educational research and the lack of reforms of the educational curricula in order to keep pace with the fundamental changes that have resulted from the decentralization of primary health care.

In addition, several other problems make this succinct analysis more complex. The unemployment rate for the country's nursing school graduates is extremely high, even for those who graduate from the best schools in Kinshasa, the capital. The situation is compounded by the almost total absence of quality management mechanisms, especially when it comes to taking stock of the health workforce that exists. The situation is a complex one in which the expected changes are not clearly discerned.

The problem of health sector human resources is vast. The context that interests us in this article is that of the human resources who are in the front line when it comes to grappling with the various communities' health problems. These are the nurses who have completed (technical) secondary school courses. They are the main primary health care professionals in Central Africa, especially in the DRC [3]. This choice limits the initial problem to a specific target population. However, the approach that we envisioned can be transferred quite easily to the other sectors concerned by health manpower management (mainly registered nurses, doctors, laboratory technicians and other health professionals).

The question of research is at three levels: on the theoretical level, this article proposes importing a theoretical model from one field to another; on the methodological level, it uses the action research-like mode of data collection to better establish results; on the empirical level, in the DRC research is unusual ground from which to introduce an innovation.

This qualitative research pursues two objectives: to present a methodology (adaptation of a health promotion model to the educational sphere) and to study the results of its application (health science teaching in the DRC). The qualitative hypothesis that subtends this thinking is that using an analytical, systemic planning and strategic synthesis model based on a systemic and participatory approach on various strategic and operational levels will procure the necessary vision for changing the basic education and training of nurses in (technical) secondary schools in the DRC.

There is a lack of literature on experiments and experiences that use analytical or planning methods to understand complex social realities and consequently the adoption of strategic plans of action that should result from such experiences. It is thus important for all the players in the process to use the outcomes of the various stages of analysis and planning to produce an appropriate and adapted logical framework. It is necessary, however, to be able to set down on paper the methodology that is used to be able to construct a model that by the end of the process appears obvious for the actors involved, i.e. teachers, internship supervisors and school management, personnel within the ministry's Directorate for Health Education and project officers.

## Methodology

Generally speaking, three relatively distinct stages were necessary, as follows:

### 1st methodological stage: set the strategic, managerial and operational levels

**Figure 1:**
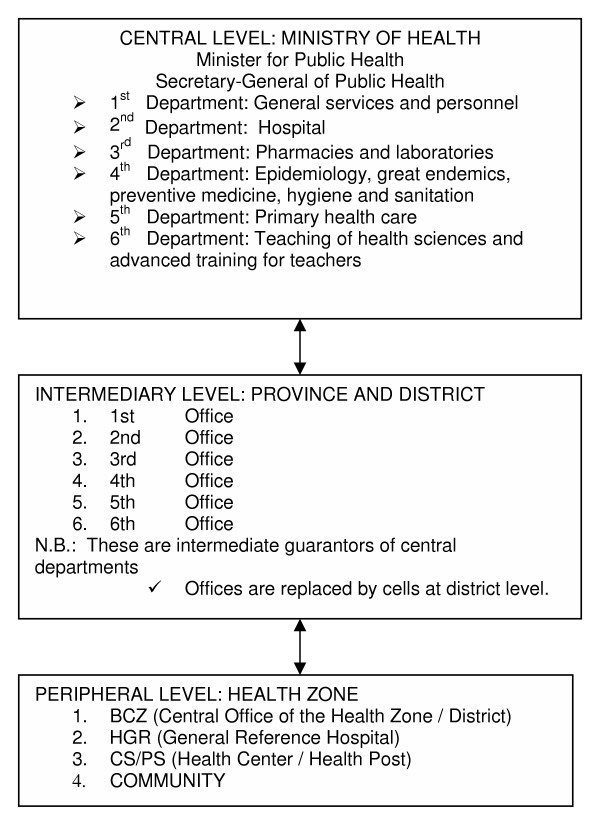
Organizational structure of the Ministry of Health of the Democratic Republic of the Congo

In the DRC, the Ministry of Health and more specifically its directorate in charge of health science education, is responsible for the basic training of secondary school nursing students and further training of health professionals (Figure 1).

The situation sometimes varies in neighbouring contexts. Thus in Rwanda, for example, the Ministry of Education is in charge of basic education and training in the technical schools and for the medical professions. In France and Belgium, the general choice was to have the Ministry of Education responsible for most of the training and education of health professionals.

We shall not discuss in this article the relevance of the place of oversight for this type of brief for training health professionals. We shall limit our remarks to the need to choose the best place for systemic planning and strategic vision in the existing context. So it is that in the 6th Directorate of the Ministry of Health of the DRC, which is in charge of paramedical secondary education, the need was perceived to develop systemic planning tools that would give a comprehensive, consistent vision of the sector's needs. While the 6th Directorate is indeed the strategic level, there was early involvement of an operational level, in the form of a sample of schools and teachers, and creation of a management unit to guide the implementation of the plans by the 6th Directorate and teachers from the grassroots.

It is important to remember that we are talking about systemic and operational planning, not just strategic planning [4]. For the strategic level, it is thus necessary to determine the organizational level that is close enough to operations on the ground, yet at the same time is independent enough to take specific, actual strategic directions.

### 2nd methodological stage: Choosing an analytical, planning and strategic synthesis model that fosters a systemic vision

This article follows on from the given that organizations and human beings are complex, and one way to have public health actions that heed this complexity is to use a systemic approach to analyse them [5].

Various models for a systemic approach exist. The approach that we chose to develop a logical framework for analysing, planning, and synthesising the work of the ministry's directorate in charge of health science education in the DRC is Green and Kreuter's PRECEDE PROCEED model [6,7]. The PRECEDE model emanates from a conceptual synthesis of the founding elements and roots of what would become health promotion.

The PRECEDE PROCEED planning model is welcome because of its multidisciplinary approach, based on the fields of epidemiology, social sciences, behavioural sciences, education and health administration. In a nutshell, the fundamental principles that gave rise to this approach come from the multifactoral nature of all problems. Once this has been posited, all efforts made to act upon behaviour, the environment and social factors must necessarily be multidimensional and multisectoral.

The acronym PRECEDE means "Predisposing, Reinforcing and Enabling Constructs in Educational/Environment Diagnosis and Evaluation", while the acronym PROCEED means "Policy, Regulatory and Organizational Constructs in Educational and Environmental Development".

The PRECEDE-PROCEED model emphasizes planning interventions by focusing on the expected outcomes of actions based on epidemiological, social, behavioural, environmental, educational, organizational, administrative and political diagnoses of a socio-health and/or educational situation. The stages in the construction of a systemic model for analysing the problem that interests us – health science teaching – are adapted as the process unfolds. One of this method's great potentials is its great flexibility, or its ability to adapt to the specific analyses' needs.

A systemic analysis and planning model is built dynamically, in a process that calls for continuous assessment. The model that the ministry's 6th Directorate came up with, and that is presented below, must change with changing knowledge in the area.

### 3rd methodological stage: allowing a participatory approach to using the model

It is important to stress the qualitative process of continual exchanges and constant observations among the players (teachers, internship supervisors, school management, ministry officers and donors) that made it possible to fill the gaps in the information-gathering process. All these elements are much more difficult to organize in one well-defined stage, but are indeed part of a process that stresses the participatory approach and comes under the third strand of the methodology being discussed. The development of the first model proceeded during the workshop held in Kinshasa at the starting of the project, with the participation of personnel from three pilot schools and the Ministry of Health in October 2002. The three-day workshop, with 40 participants from various institutional levels, permitted the establishment in December 2003 of strategic orientations and guidelines for the continued broadening of the programme.

All PRECEDE PROCEED models are built upon the players' actual experiences of the problem to solve. The clarification of the problem itself, which is part of the epidemiological and social diagnosis, comes out of a debate that must be conducted with all the parties concerned. This problem will gradually become more and more clear as its statement shuttles back and forth among the parties until it eventually satisfies the strategic and institutional level that is in charge of the programme and that the problem concerns directly. If, thanks to a resolutely participatory approach, all of the players adopt the use of a systemic approach on a real strategic, managerial and operational level, it will become a solid tool for the entire teaching body concerned.

## Results and discussion

The presentation of results is at two levels: PRECEDE results and PROCEED results. The first are mainly descriptive (to tell and analyse the facts). PROCEED results are more normative, leading to certain recommendations for practitioners and other actors.

### PRECEDE results

To facilitate presentation of the results and understanding of this coherent, overall vision of the interrelated elements, it was considered pertinent to retranscribe the full model as it exists for the 6th Directorate of the DRC's Ministry of Health.

To structure the results' presentation, we shall follow the order in which the model's construction progressed. The table must be read from right to left, starting with the epidemiological and social diagnosis, then going on to the behavioural diagnosis and from there to the analysis of the educational and environmental determinants of these behaviours, and then to end with the analysis of the institutional diagnosis (see Additional file 1).

### Epidemiological and social diagnosis

In the Ministry of Health, all the players are concerned by the mortality and morbidity indicators in the country. For health professionals, the lack of quality of the service provision and care provided by their health system is an obvious cause of the people's lack of confidence in their health system [8]. However, to produce a verifiable systemic analysis and then effective strategic synthesis within the directorate that interests us, the problem of the directorate in charge of health science education must first be clarified in connection with this broader problem. Thus the mismatch between health science teaching and the competence that health professionals are expected to have was seen as connected to the lack of quality in health care and services. All the players on their various organizational levels – ministry staff, teachers, basic supervisors, donors and project officers – took this diagnosis on board as a major concern.

### Behavioural diagnosis

Who are these players and what behaviour can explain, through a direct link, the diagnosis of inadequacy? In answering these questions with the players themselves, we discover that there are groups of players that are never clearly identified yet are clearly related to this problem of inadequacy. This is the case, for example, of the donors and nongovernmental organizations (NGOs).

Revealing all the groups of players makes it possible to see more easily why importance should be given to a multisectoral approach, especially one that covers teachers and medical and paramedical professionals. If the population is considered a group of players that is separate from the problem at hand, it will not be possible to take it into account in setting up action strategies, to the extent that the aim of such work is to better define people's expectations in terms of the quality of care and arrive at a better understanding of their behaviour.

During the discussions, the teachers felt that priority had to be given to separating the group of teachers from that of intern supervisors in order to better highlight the particularities and role of the field training. The school managements revealed their specific role in this problem of mismatches. Indeed, the teachers' and supervisors' behaviour is strongly linked to their own behaviour in dealing with changes [9]. We have presented one or the other behaviour for each of these groups of players as examples only.

### Environmental diagnosis

This diagnosis allows for the factors that are linked to the environment and are direct causes of the epidemiological and social diagnosis. In a context such as that of the DRC, geopolitical and socioeconomic factors head the list, along with the health structures' inaccessibility. To take a more constructive approach without denying reality, it is necessary to focus the analysis of this diagnosis on the more targeted problem of the inadequacies in the training sector. This reveals variables that are more controllable for the levels that are concerned and that everyone agrees are connected to the problem. These are: the learning environment, teaching environment, class hours that facilitate or hamper certain types of learning, etc.

### Educational and motivational diagnosis

The educational diagnosis enables one to home in on the educational and motivational determinants, which must not be overlooked when one goes on to an interventional phase. To the extent that the systemic approach gives significance to each group of players (teachers, learners and others) as well, as is the case in the DRC, it is fully possible to set up a frame of analysis, assessment and action-research that presents the variables and determinants in a PRECEDE model that are specific to each specific group (action-research framework). This is what was done in the DRC to follow the changes in teaching practices, in conjunction with each intervention that was identified, that were made in the specific group of teachers. The results show that it is relatively easy to separate the educational determinants from each other in order to facilitate subsequent reflection about the strategic action to take.

The predisposing factors that concern knowledge, experience, attitudes, perceptions and representations have a key place in relation to the behaviours of the players of interest to the Directorate for Education. This construction shows clearly that the training given is usually concerned with knowledge only and generally does not make use of the learners' life experiences.

The other important result is to be able to visualize the place of representations in a conceptual framework that will likewise be used for the action. For example, there are the various representations of learning theories when it comes to teaching methods or unfounded beliefs about the quality of care. Specific models exist that enable one to delve much deeper into perceptions and beliefs [10,11]. These are complementary research models. When we are seeking to develop a tool that can be used to construct an operational model for strategic choices of action to take on a high institutional level, the possibility of providing this place for representations and beliefs is already vital and elucidating.

The enabling factors in terms of actual competences (skilfulness, know-how and behaviour) are too often disregarded and underestimated in interventions. Incorporating them in this model thus enables the directorate in charge of this branch of education to check to what extent the projects, programmes and other support measures consider this priority strand in terms of development independence.

The reinforcing factors, which are sometimes also referred to as facilitating factors, are the determinants that act upon the positive feedback loops. The importance that all the players give to this type of variable in constructing the model confirmed the need that the directorate had already felt to find means to set up long-range monitoring mechanisms for the various activities engendered by the programme or by some more specific projects.

The model contains a certain number of variables. It is clear that it can be enriched in the course of the process through its use and the players' better discovery and gradual appropriation of its features.

### Institutional diagnosis

In terms of results, the institutional diagnosis requires analysing the situation at the organizational level that corresponds to the level of the model's application. This is a national health science education programme under the Ministry of Health. As such, the institutional diagnosis stresses essential strategic variables if one wants to work on a well-knit, comprehensive set of changes. It thus entails the need to analyse the institutional standards when it comes to inspections and assessment, but also those governing health system management and health sector human resource quality management (for example, the existence or lack of a Nursing Board). This is also the level on which we shall discuss how the programme dovetails with other variables and determinants.

### PROCEED results

Before strategic thinking can be put into place based on this situational analysis, it is possible to go on to a more dynamic reading of the relations between determinants and variables. So it is that the DRC's Ministry of Health directorate in charge of health science education foresees a certain number of strategic axes for action. The aim of the action is the problem's translation into an objective form. The directorate thus considered its main goal to be to improve the match between what is learnt in schools and health professionals' needs and the population's expectations.

**Figure 2:**
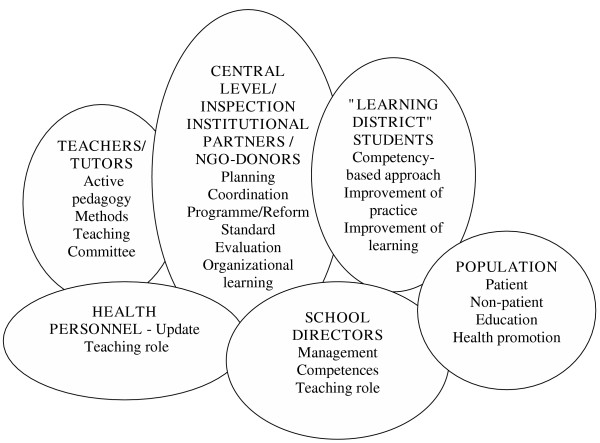
Visual summary of considered actors

To better understand how the reading of the conceptual model brought us to the action strategies, it is useful to stress an intermediate step that is summarized in Figure 2.

A natural adaptation of the PRECEDE model was to define the groups of players by their behaviour. In this way, we obtained a better picture of the division of responsibilities to achieve a common goal and evidence of the need for interdisciplinary work [12]. A comprehensive reading of the PRECEDE model points us towards a strategic choice that integrates an institutional and educational approach from education with an epidemiological and social approach from health and welfare. The players in their respective environments are located between the two. This model shows the need to find a common thread between education and health needs that allows for the place and role of each group of players in their context.

The results bring to the fore a number of behaviours that attest to a lack of independence, absenteeism, lack of collaboration, failure to connect theory and practice, a lack of communication, ignorance of the teacher's role, etc., depending on the group. Examination of these results prompts us to stress the importance that must be given to the learning environment and, when it comes to action strategies, the importance to give to a learning environment that is in tune with the strategic axes that are selected, in this part just as in the other parts of the situation's analysis.

Given this finding and the need to link the educational and institutional diagnosis with the health problem (seen as an appropriate form for education), one proposed strategic hypothesis is to favour learning techniques that are based on active teaching methods [13].

In going consistently through the various diagnoses and organizational levels, this choice led the education directorate to think about changing its programmes and standards so as to base them on novel teaching concepts such as skills-based learning [14] and setting a skills reference framework on the basis of in-depth research done with the entire set of clearly identified target populations.

The reading of these results in terms of strategic action reveals the need to bolster the analytical and planning process that already exists within the directorate to pay more attention to the educational and environmental determinants for all the target populations concerned. To our mind, the success of the expected changes in terms of narrowing the gap between the "supply" and the "demand" hinges on this.

**Figure 3:**
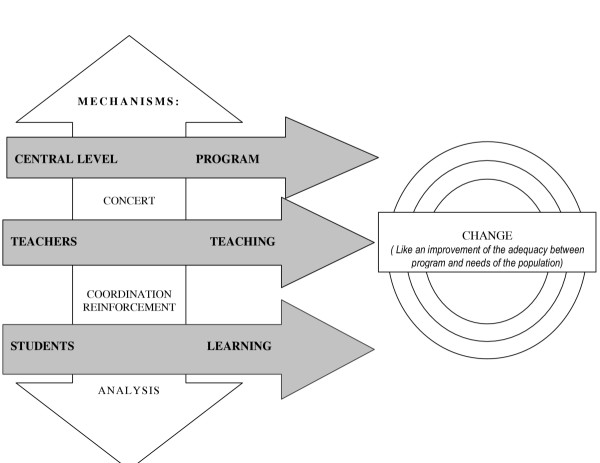
Strategic axes for action

The following diagram summarizes the strategic axes that the Directorate for Health Science Education chose to achieve this objective on the basis of the PRECEDE analysis (Figure 3).

This figure reveals four axes to be reinforced:

• to reinforce communication and coordination in conjunction with the other reinforcing factors: the pilot schools' teaching method committees, teaching monitoring and feedback, the setting-up of networks, etc.;

• to develop methods to enhance the learner's autonomy: active teaching, constructivist approach, interdisciplinary, critical spirit, etc.;

• to foster a learning environment that enables the learners to acquire knowledge: library, teaching materials, computer learning, computerized documentation centre, etc.;

• to provide institutional and structural support: standards and curricula in tune with teaching and organizational innovations and skills targets that fit health professionals' needs and meet the community's expectations.

The discussion will take place on two levels – the operational and the conceptual. On the operational level, we feel it is interesting not to dwell on the presentation of the model as it could have been applied, but on its actual application. The results are presented so as to allow the reader to understand how to organize the problems that are felt to exist in health science education in the DRC.

Even if the Directorate for Health Science Education is well aware of its problems, the systemic modelling of the interconnected variables and populations seems to give it a conceptual and operational tool that is useful on various levels, as follows:

• tool for dynamic analysis of the situation with regular updates;

• tool for systemic planning that also enables the directorate to put forward arguments in dealing with donors and NGOs in the sector;

• assessment tool that gives more importance to assessment criteria such as cohesiveness, consistency, relevance, appropriation, and comprehensiveness, i.e., process criteria;

• research and evaluation tool that can also promote a more quantitative approach to analysing the relations between variables and various diagnoses or within the same diagnosis;

• a dialogue-enhancing tool, for it gives the groups of players involved a vision of the planned change and a common objective.

To sum up, this is a tool that provides a certain guarantee that the strategy development process is informed, meets the needs and is complete [15].

The list of these advantages is obviously based on some baseline conditions: a participatory process in which the model is developed and operates and the appropriation of the concepts that subtend the model [16]. Even though it was more difficult to describe how the intervention strategies are set, based on the construction of this model, we should like to stress that a complete analysis of the situation that is based on this systemic approach usually reveals the relevant strategic axes on its own and despite the protagonists' limited ability to synthesize the situation.

**Figure 4:**
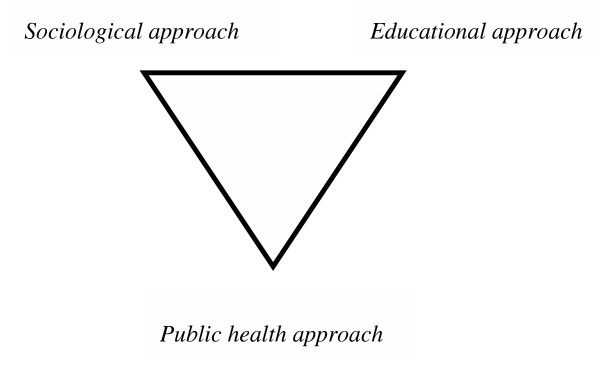
Multi-field vision of the change

On the conceptual level, the discussion will revolve around Figure 4.

We observed through the PRECEDE analysis and then the PROCEED strategic reflection phase that many disciplines converged in order to lead us to this hypothesis and a common objective of needs-matching. Indeed, when action is carried out it will be a matter of achieving a gradual advance that occurs along the (horizontal and vertical) strategic axes defined earlier in this article. Moreover, we are confronted with strategic choices that involve at least three dimensions: a public health approach, an education approach and a sociological approach.

These three dimensions are part of the data collection process's success, as well as the success of the strategic choices that follow. This reinforces the fact that the PRECEDE PROCEED model comes from the development of an approach aimed at meeting the need for education and health promotion tools and methods. So it is that we see numerous applications of this process in technical health education establishments that spring from a true systemic analysis of the problems with full mastery of a structuring capacity, unlike some other models such as causal analysis (17).

Similarly, we can consider that defining a problem at an institutional and organizational level also requires the identification and involvement of all the parties concerned. We can also consider that the tools that help to understand the relations between elements and insist on a better search for behavioural determinants are prerequisites for organizational learning that has groups of players interact with each other. This is all the more true if the change that is ultimately expected (a match between two sides of the equation) is contingent on changes in the players' behaviour and practices, as is the case of health education.

In terms of limits of this research, it targets the analysis of an inadequacy within human resources' management in health, which is that of training of nurses from professional technical levels. Other levels of inadequacies are worthy to be analysed in a complementary way relating to other health professionals, the sectors of health and education planning. The Green model is complementary to the use of methodological dynamic references much as the management of the project cycle focuses on managing interventions or projects whose aim is to contribute to changing a situation from unsatisfactory to satisfactory. Its use within the framework of the project could obtain more means while enabling developments relating to action research. In this context, the contribution from other disciplines, such as psychology, could be reinforced.

## Conclusions

With regard to the three levels of starting research – the theoretical, methodological and empirical – PRECEDE PROCEED analysis is a model that can be applied to varied situations and problems, although it must be used participatively and proactively in order to enhance its utility in specific circumstances as a personal transfer tool. On the empirical level, the will of all actors – and the Ministry of Health in particular – to have a clear vision of the projected change and manner of reaching that point, while integrating the complexity, was the element carrying the process. We advance the hypothesis that L. Green's systemic approach may become one of a set of active methods, such as problem-based learning, cooperative learning, or even project-based learning, to transfer to learners in nursing schools and sections in the DRC. Indeed, the ability to analyse and synthesize, but also to carry out education and health promotion actions, is essential.

## List of abbreviations

DRC: Democratic Republic of Congo

PRECEDE: Predisposing, Reinforcing and Enabling Constructs in Educational/Environment Diagnosis and Evaluation

PROCEED: Policy, Regulatory and Organizational Constructs in Educational and Environmental Development

## Competing interests

The authors declare that they have no competing interests.

## Authors' contributions

FP is responsible for this research. She initiated the project in DRC. She is a specialist in public health and pedagogy. She participated in the design and coordination of the study and drafted the manuscript.

JK and DB are two teachers in charge of the reform of the nursing programme in DRC. They set up the collection of data for this study and finalized the analysis.

YC and AL participated in the design of the study and the adaptation of de Green's model in the field of nursing training. YC participated in writing the manuscript.

MG specializes in management and pedagogy. She conceived the study with FP and participated in its design. DP took part in the elaboration of the methodology. She brought an expertise in health promotion and wrote part of the manuscript.

All authors read and approved the final manuscript.

## Supplementary Material

Additional File 1 Table 1. PRECEDE model for health science teaching in the DRC oversized table Click here for file
