# Supplementary material for: A model for analysis, systemic planning and strategic synthesis for health science teaching in the Democratic Republic of the Congo: a vision for action
Source: Hum Resour Health. 2004 Dec 7;2:16. doi: 10.1186/1478-4491-2-16 (PMC543448; doi:10.1186/1478-4491-2-16)
Supplement: Additional File 1 — Table 1. PRECEDE model for health science teaching in the DRC oversized table [file 1478-4491-2-16-S1.pdf]

Table 1. PRECEDE model for health science teaching in the DRC

Educational diagnosis

Predisposing factors
Knowledge: Existing (experience); multidisciplinary (learning theories); pertinence (theoretical and practical needs); professional techniques; evidence-based education/information…
Attitude: Ready for a radical move in concept (changing paradigm); favourable to appropriate and adapted technologies
Perception: of one's role (pedagogic, medical, professional…)
Believes: representations of health problems

Capacitating factors
Individual access to ongoing training; individual access to databases; ability in techniques of care; ability in learning techniques; individual adaptation of methods and tools; capacity and competence in situation of multiple common problems; critical analysis; analysis of one's practices; research capacities; capacity in

Reinforcing factors
- Teaching follow-up and mechanisms of individual and collective feedback
- Teamwork
- Life experience
- Network of schools in nursing sciences

Behavioural diagnosis: *Behavior of the actors*

Population:
- Participate in the management of its health system
- Adopts democratic behaviour

**** Teachers, tutors:***
- Link theory and practice to field reality
- Update their course content and methods
- Do not update knowledge in their lectures

**** Tutors:***
- Propose a rigorous framing

**** School directors:***
- Support sufficiently the continuing education of teachers

Health personnel:
- Involve the population

Schools / students:
- Pupils often absent
- Pupils do not learn autonomously

**** Central administration :***
- Does not collaborate well

**** Donors, NGO:***
- Work independently from political circumstances
- Work coherently with global objectives and not only for their own priorities

Social and epidemiological diagnosis

Problems :

- Mortality indicators
- Morbidity indicators

- Lack of quality in health services and care

- Loss of confidence of the population in its health services (low acceptability)

- **Gaps between their basic training, expectations of the population and health professionals' actual training needs**

Environmental diagnosis

- Health staff salaries, teachers' salaries …
- Quality of the infrastructure (medical, training, housing…)
- Lack of teaching and medical equipment
- Collective access to ongoing training and databases
- High number of pupils
- War and political instability

…

Institutional diagnosis

School project – Percentage of budget assigned to health – Strategic orientations of the health policy and operational planning – Coherence of programs and evaluation with the expected capacities of human resources – Existing legal body, such as a nursing council, to ensure quality control – Institutional transparency between professionals: doctors, nurses, teachers – Norms coherent with the expected change – Career scheme in relation to job descriptions and required qualifications – Existing mechanisms for intersectorality or multidisciplinary approach – Lack of data bases on human resources
